# Influence of Mass and Social Media on Psychobehavioral Responses Among Medical Students During the Downward Trend of COVID-19 in Fujian, China: Cross-Sectional Study

**DOI:** 10.2196/19982

**Published:** 2020-07-20

**Authors:** Yulan Lin, Zhijian Hu, Haridah Alias, Li Ping Wong

**Affiliations:** 1 Department of Epidemiology and Health Statistics, Fujian Provincial Key Laboratory of Environment Factors and Cancer School of Public Health Fujian Medical University Fuzhou China; 2 Centre for Epidemiology and Evidence-Based Practice Department of Social and Preventive Medicine, Faculty of Medicine University of Malaya Kuala Lumpur Malaysia

**Keywords:** psychobehavioral, COVID-19, mass media, social media, medical students, China

## Abstract

**Background:**

An extensive amount of information related to the novel coronavirus (COVID-19) pandemic was disseminated by mass and social media in China. To date, there is limited evidence on how this infodemic may influence psychobehavioral responses to the crisis.

**Objective:**

The aim of this study is to assess the psychobehavioral responses to the COVID-19 outbreak and examine their associations with mass and social media exposure.

**Methods:**

A cross-sectional study among medical and health sciences students from the Fujian Medical University in Fuzhou, China, was conducted between April 6-22, 2020.

**Results:**

A total of 2086 completed responses were received. Multivariable analyses demonstrated that four constructs of the Health Belief Model (HBM)—higher perception of susceptibility (odds ratio [OR] 1.44; 95% CI 1.07-1.94), severity (OR 1.32; 95% CI 1.10-1.59), self-efficacy (OR 1.61; 95% CI 1.21-2.15), and perceived control or intention to carry out prevention measures (OR 1.32; 95% CI 1.09-1.59)—were significantly associated with a higher mass media exposure score, whereas only three constructs—higher perception of severity (OR 1.43; 95% CI 1.19-1.72), self-efficacy (OR 1.85; 95% CI 1.38-2.48), and perceived control or intention to carry out prevention measures (OR 1.32; 95% CI 1.08-1.58)—were significantly associated with a higher social media exposure score. Lower emotional consequences and barriers to carry out prevention measures were also significantly associated with greater mass and social media exposure. Our findings on anxiety levels revealed that 38.1% (n=795; 95% CI 36.0-40.2) of respondents reported moderate-to-severe anxiety. A lower anxiety level was significantly associated with higher mass and social media exposure in the univariable analyses; however, the associations were not significant in the multivariable analyses.

**Conclusions:**

In essence, both mass and social media are useful means of disseminating health messages and contribute to the betterment of psychobehavioral responses to COVID-19. Our findings stress the importance of the credibility of information shared through mass and social media outlets and viable strategies to counter misinformation during a pandemic.

## Introduction

With rapid increases in the number of internet users, both mass and social media have a prominent role to play in modern society. In China, there were approximately 688 million internet users, of whom 75.1% were aged 10-39 years, in 2015 [1]. As the general public becomes more health conscious, the popularity of social media as a means of acquiring health-related information has been growing in recent years [2,3]. Of note, social media tools are readily accessible on the internet and have become even easier to access via apps on smartphones. As a result, the role of social media as a pathway to news is very popular [4]. However, social media users may be exposed to untrustworthy news or information of questionable accuracy. Inaccurate information acquisition could have detrimental effects, since passive acquisition through social media, particularly through WeChat Moments, is an important medium for health information acquisition among college students in China [2]. (WeChat is the most popular social media platform in China and includes instant messaging and service platforms to carry out payment, marketing, and promotion activities. WeChat Moments is an interactive platform that allows users to share information/news articles, photos, and video.) Moreover, almost 60% of social media users admitted that internet-based health information impacted their health management strategy [5]. Mass media, in contrast, provides more credible information and has been used as a means of communication of scientifically accurate information about health more often than social media. Mass media can influence health behaviors and promote health behavior change in the public [6].

In late December 2019, an unknown form of pneumonia—caused by a novel coronavirus—surfaced in Wuhan, China, and rapidly spread across the globe. By the end of April, the overall number of the coronavirus disease (COVID-19) cases worldwide increased to 2,878,196 and the death count reached 198,668 [7]. China, after over 3 months of battling COVID-19, has managed to control the outbreak. Nonetheless, the community at large in China remains vulnerable, and prevention from rebound is essential since lockdown regulations have been relaxed. During the early phase and the peak of the COVID-19 epidemic in China, various issues surrounding mental distress among the general public caught the attention of researchers. Studies showed that a great proportion of the general public was found to have severe depressive symptoms, even during the early phase of the outbreak [8,9]. It is important to address mental health issues during a disease outbreak, as it may weaken social and other areas of functioning, including an impairment in prevention measures [10,11]. Psychobehavioral responses have been understudied after the cessation of the COVID-19 outbreak in China and this warrants attention. The lay public's psychobehavioral responses during a disease outbreak play an important role in bringing the outbreak under control [10]. Hence, to avoid a resurgence of infections, investigation into preventive behavioral responses in addition to the psychological well-being of the public post–COVID-19 warrant attention. Attitude is a key factor that determines behavioral intention. The Health Belief Model (HBM) has been used as the theoretical framework to explain the health behaviors of individuals. It includes the following concepts: perceived susceptibility, perceived severity, perceived benefits, perceived barriers, cues to action, and self-efficacy [12,13]. Adopting the HBM to explain psychobehavioral changes during the COVID-19 outbreak is essential.

One study in China, conducted during the early phase of the outbreak, found a high prevalence of mental health problems among the public, which was positively associated with frequent exposure to social media [14]. To the best of our knowledge, little research has been conducted on social or mass media exposure now that China has entered the downward trend of COVID-19 transmission. Thus, an investigation of exposure to both mass and social media and linkage to the psychobehavioral health outcomes of the public is needed. Accurate information-seeking behaviors during the COVID-19 outbreak has important implications for health-related behavior change and may strengthen infection prevention and control. The traditional mass media is message-driven; in contrast, social media is conversation-driven, and during the COVID-19 outbreak, it is unclear which form of media influences the public and shapes their psychobehavioral responses. Therefore, this study aimed to (1) assess the level of mass and social media exposure related to COVID-19 and (2) identify the association between both forms of media exposure with HBM constructs, psychological and behavioral responses, and anxiety levels.

## Methods

### Participants and Study Design

An anonymous internet-based, cross-sectional, open survey was distributed to medical and health sciences students at Fujian Medical University, Fuzhou, China, between April 6-22, 2020. Convenience sampling was used to recruit subjects for this study. The link to the survey questions was sent to administrators or lecturers of all departments to be disseminated to registered students at the university. In an attempt to reach comprehensive recipient coverage, the link to the survey was also sent to students’ social media groups and forums. All respondents were informed that their participation was voluntary, and consent was implied through their completion of the questionnaire. No incentives were provided to the study participants.

The questionnaire was developed in English, then translated into Chinese. Local experts performed face validation on the content of the questionnaire. The online questionnaire was subsequently pilot tested for readability and clarity of items on 30 participants from the general public. A minor revision was made based on the results of the pilot study. The revised questionnaire was further pretested before field administration. The survey consisted of questions that assessed demographic background, mass media and social media exposure, constructs from the HBM, psychological and behavioral responses, and anxiety levels associated with the COVID-19 outbreak.

### Instruments

#### Mass Media and Social Media Exposure

Questions on mass media (8 items) and social media (10 items) exposure queried participants about types of information acquisition. The response options were scored on a 4-point Likert scale (0=never, 1=rarely, 2=sometimes, and 3=often). The scores were summed, with higher scores representing higher usage. The possible score range for mass media exposure and social media exposure was 0-24 and 0-30, respectively. The participants were informed that the term “mass media” refers to both traditional and online mass media (written or broadcast), including television, radio, advertising, newspapers, magazines, and newsfeeds. In contrast, “social media” refers to websites and apps such as WeChat, Weibo, and Youku, which are among the most commonly used social media platforms in China. Weibo shares features similar to Twitter (eg, allows users to share content up to a 140-Chinese-character limit). On the other hand, Youku, often called the YouTube of China, is an online video and streaming service platform.

#### HBM Constructs

Questions related to HBM constructs include perceived severity, perceived susceptibility, perceived efficacy, and perceived control or intention [12,13,15]. Perceived severity was measured using a 1-item question (*How serious do you think COVID-19 is?*) on a 4-point scale (*not at all serious* to *very serious*). Perceived susceptibility was a 1-item question (*What do you think are your chances of getting COVID-19?*) on a 4-point scale (*not at all* to *very large chance*). Perceived efficacy was measured using a 1-item question (*Do you think that you will manage to carry out prevention measures currently recommended by the authorities?*) on a 4-point scale (*certainly cannot* to *most certainly yes*). Perceived control or intention was measured using a 1-item question (*Would you carry out prevention measures currently recommended by the authorities?*) on a 4-point scale (*certainly cannot* to *most certainly yes*).

#### Psychological and Behavioral Responses

Psychological responses measure the emotional consequences of the COVID-19 outbreak. The emotional consequences consist of questions about feelings of fear, avoidance, keeping a secret, embarrassment, and stigma associated with COVID-19 (5 items). Optional answers were on a 4-point Likert scale, with the items scored as 1 (strongly disagree), 2 (disagree), 3 (agree), or 4 (strongly agree). The possible total emotional consequences score ranged from 5-20, with higher scores representing higher levels of emotional consequences.

Behavioral response measures relating to preventive barriers consist of 5 sections (8 items) that comprise questions about personal protection (3 items), cough etiquette (3 items), and contact precautions (2 items). The question queried participants’ level of difficulty in practicing physical prevention measures. A 4-point Likert scale was used to report responses, with scores of 1 (very easy), 2 (easy), 3 (difficult), or 4 (very difficult). The total physical prevention barriers score ranged from 8-32, with higher scores representing higher difficulty levels of physical prevention.

#### Anxiety

Anxiety was measured using the 6-item state version of the State-Trait Anxiety Inventory (STAI-6) [16,17]. The respondents rated the frequency of experiencing 6 emotional states (ie, being calm, tense, upset, relaxed, content, and worried) as a result of the COVID-19 outbreak. A 4-point scale was used (ie, 1=not at all, 2=somewhat, 3=moderately, and 4=very much). The scores on the 3 positively worded items were reverse-coded. The total summed scores were prorated (multiplied by 20/6) to obtain scores that were comparable with those from the full 20-item STAI (giving a range of 20-80) [17]. A cut-off score of 44 was used to indicate moderate-to-severe symptoms [10,18].

### Statistical Analysis

The reliability of the scales used was evaluated by assessing the internal consistency of the items representing the scores. The mass media and social media exposure items had a reliability (Cronbach α) of 0.958 and 0.940, respectively. The emotional consequences and prevention barrier behavior items had a reliability (Cronbach α) of 0.794 and 0.840, respectively. The reliability computed for the STAI-6 items in the assessment of anxiety was 0.793.

Multivariable logistic regression analysis, using a simultaneous forced-entry method, was used to determine the factors influencing mass media and social media exposure. Multivariable logistic regression analyses were performed on all variables found to have a statistically significant association (two-tailed, *P*<.05) in the univariable analyses. Odds ratios (ORs), 95% CIs, and *P* values were calculated for each independent variable. All statistical analyses were performed using SPSS, version 20.0 (IBM Corporation). The level of significance was set at *P*<.05.

### Ethical Considerations

This research was approved by the Research Ethics Committee of the Fujian Medical University. Written informed consent was not acquired from participants. The committee approved that consent was implied through questionnaire completion and submission.

## Results

**Figure 1:**
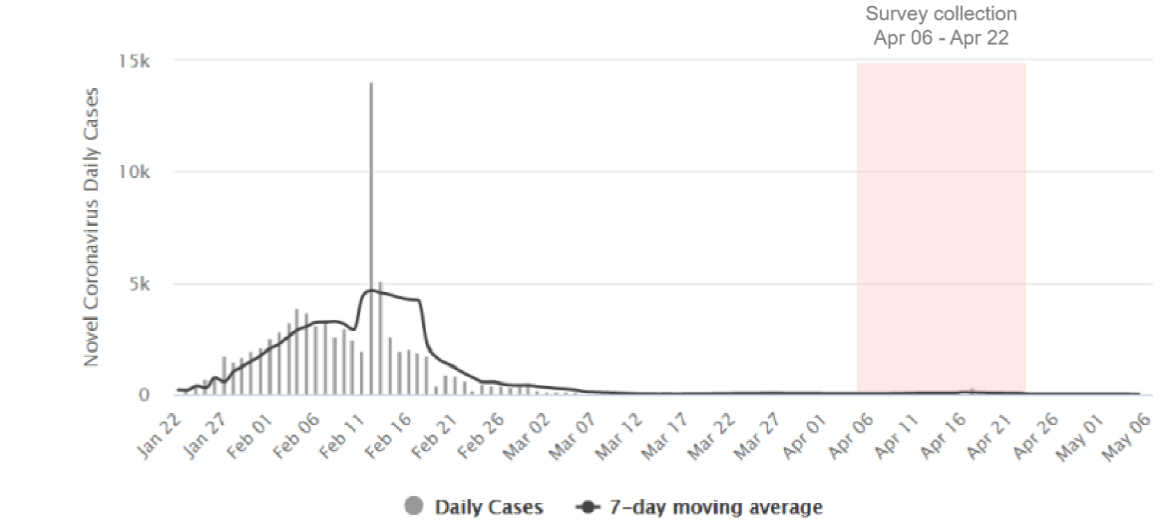
Daily new cases in China since the beginning of the coronavirus disease (COVID-19) outbreak and throughout this study's data collection period.

A total of 2086 completed responses were received. Figure 1 shows the number of daily new cases in China since the beginning of the COVID-19 outbreak [19] and the duration of our data collection period. As shown in Figure 1, data collection was carried out past the peak of the COVID-19 outbreak.

As shown in Table 1, more than half of the participants were 18-20 years old (n=1197, 57.4%). Nearly two-thirds of the birthplaces of participants were in rural areas (n=1369, 65.6%). Most participants reported that their annual family income was below CNY 50,000 (n=978, 46.9%) or in the CNY 50,000-120,000 category (n=775, 37.2%). The distribution by university year was approximately equal.

**Table 1:** Demographic characteristics of participants (N=2086).

Characteristic			Count, n (%)
**Age group (years)**			
	18-20		1197 (57.4)
	21-22		714 (34.2)
	23-29		175 (8.4)
**Birthplace**			
	Urban		717 (34.4)
	Rural		1369 (65.6)
**Annual family income (CNY)**			
	<50,000		978 (46.9)
	50,000-120,000		775 (37.2)
	>120,000		333 (16.0)
**Year**			
	1		662 (31.7)
	2		490 (23.5)
	3		606 (29.1)
	4 and postgraduate		328 (15.7)
**Health Belief Model**			
	**Perceived susceptibility**		
		Certainly no/probably no/probably yes	2001 (95.9)
		Certainly yes	85 (4.1)
	**Perceived severity**		
		Not at all/slightly serious/serious	1101 (52.8)
		Very serious	985 (47.2)
	**Perceived self-efficacy**		
		Certainly no/probably no/probably yes	580 (27.8)
		Certainly yes	1506 (72.2)
	**Perceived control or intention to carry out preventive measures**		
		Certainly no/probably no/probably yes	788 (37.8)
		Certainly yes	1298 (62.2)
**Psychological and behavioral response**			
	**Emotional consequences**		
		Scores 5-9	1004 (48.1)
		Scores 10-20	1082 (51.9)
	**Barriers to carry out preventive measures**		
		Scores 8-15	986 (47.3)
		Scores 16-32	1100 (52.7)
**Anxiety level**			
	**State-Trait Anxiety Inventory**		
		Scores 20-43	1291 (61.9)
		Scores 44-80	795 (38.1)

### Mass Media and Social Media Exposure

**Figure 2:**
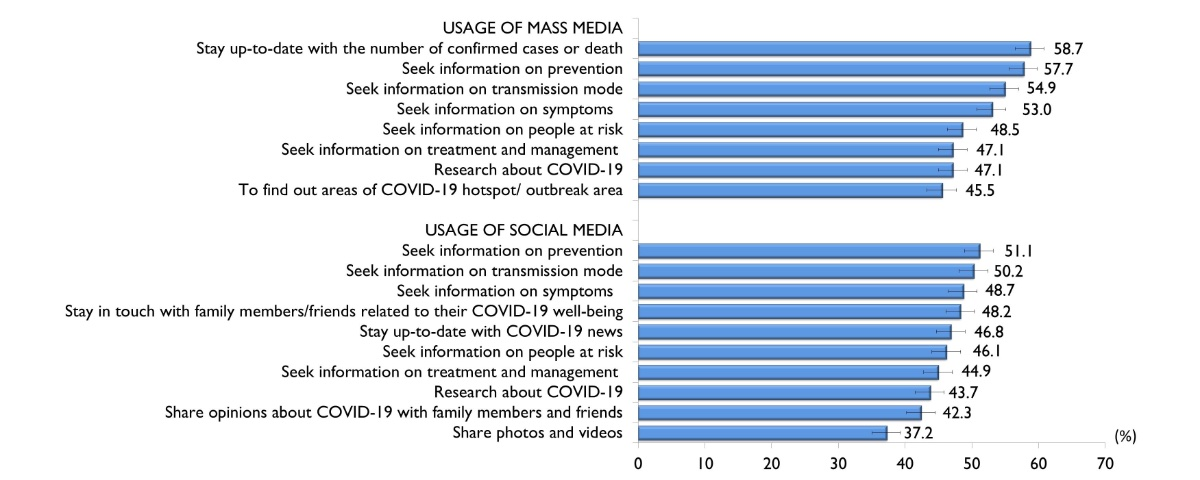
Proportion of participants who "often" used mass media and social media (N=2086).

Figure 2 shows the proportion of *often* responses and its corresponding 95% CIs for mass and social media use. The majority of participants relied on mass media for staying up-to-date with information about the number of confirmed COVID-19 cases or deaths (n=1224, 58.7%), followed by information seeking related to prevention (n=1204, 57.7%), transmission (n=1145, 54.9%), symptoms (n=1105, 53%), and risk (n=1012, 48.5%) associated with COVID-19. The most common reasons to use social media were to obtain information about prevention (n=1065, 51.1%), transmission (n=1048, 50.2%), and symptoms (n=1015, 48.7%) of COVID-19.

The mean total mass media exposure was 19.3 (SD 4.9; range 0-24) out of a possible score of 24. The median was 20.0 (IQR 16.0-24.0). The total mass media exposure scores were categorized into two groups (20-24 or 0-23), based on the median split; as such, a total of 1113 (53.5%; 95% CI 51.2-55.5) were categorized as having a score between 20-24 and 973 (46.6%; 95% CI 44.5-48.8) had a score between 0-23. The mean total social media exposure was 23.2 (SD 5.8; range 0-30) out of a possible score of 30. The median was 23.0 (IQR 20.0-29.0). The total social media exposure scores were categorized into two groups (23-30 or 0-22), based on the median split; as such, a total of 1096 (52.5%; 95% CI 50.4-54.7) were categorized as having a score between 23-30 and 990 (47.5%; 95% CI 45.3-49.6) had a score between 0-22.

### HBM Constructs

In total, 1558 participants (74.7%; 95% CI 72.8-76.5) reported *certainly yes*/*probably yes* for perceived susceptibility of getting infected with COVID-19. A relatively lower proportion perceived COVID-19 as very serious (n=985, 47.2%; 95% CI 45.1-49.4). The majority also reported *certainly yes* (n=1506, 72.2%; 95% CI 70.2-74.1) in their ability to carry out recommended prevention measures. A relatively lower proportion reported *certainly yes* (n=1298, 62.2%; 95% CI 60.1-64.3) about their intentions to carry out the recommended prevention measures.

### Psychological and Behavioral Responses

**Figure 3:**
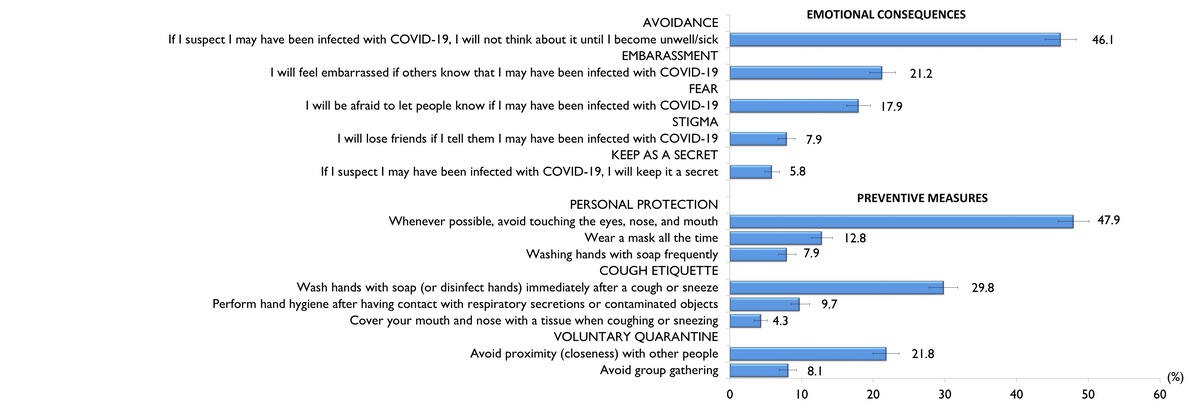
Proportion of participants who answered "agree/strongly agree" for questions related to emotional consequences and "difficult/very difficult" for questions related to carrying out preventive measures (N=2086).

Figure 3 shows the proportion and corresponding 95% CIs of responses for items on emotional consequences. Nearly half of the participants answered *strongly agree*/*agree* in regard to avoidance behavior (n=962, 46.1%); 21.2% (n=443) and 17.9% (n=374) strongly agreed or agreed that they felt embarrassment or fear, respectively. The mean total emotional consequences score was 9.4 (SD 2.7; range 5-20). The median was 10 (IQR 7-11). The total emotional consequences scores were categorized into two groups (10-20 or 5-9), based on the median split; as such, a total of 1082 (51.9%; 95% CI 49.7-54.0) were categorized as having a score between 10-20 and 1004 (48.1%; 95% CI 46.0-50.3) were categorized as having a score between 5-9.

The proportions of *difficult/very difficult* responses and the corresponding 95% CIs for difficulties in carrying out preventive measures are also shown in Figure 3. The greatest difficulty reported was avoiding touching one’s eyes, nose, and mouth (n=1000, 47.9%). Difficulties in avoiding proximity with other people and wearing a mask all the time were also reported by 21.8% (n=454) and 12.8% (n=267) of participants, respectively. The mean total score for barriers to carry out preventive measure was 15.0 (SD 3.7; range 8-32). The median was 16 (IQR 12-17). The total score for barriers to carry out preventive measures was categorized into two groups (16-32 or 8-15), based on the median split; as such, a total of 1100 (55.7%; 95% CI 50.6-54.9) were categorized as having a score between 16-32, and 986 (47.3%; 95% CI 45.1-49.4) were categorized as having a score between 8-15.

### Anxiety

The mean overall anxiety score was 40.4 (SD 10.8; range 20-80). Using a cut-off score of 44 for the STAI score, a total of 38.1% (n=795) (95% CI 36.0-40.2) of participants reported moderate-to-severe anxiety (score=44-80). Participants in the 18-20 years age group (n=477, 39.8%) reported the highest amount of moderate-to-severe anxiety, followed those who were 21-22 years old (n=270, 37.8%) and 23-29 years old (n=48, 27.4%) (χ^2^_2_=10.027, *P*=.007). There was a gradual decrease in the proportion of moderate-to-severe anxiety by university year, whereby 41.4% (n=274) of year 1 participants reported moderate-to-severe anxiety compared to 40.0% (n=196) among year 2, 39.8% (n=241) among year 3, and only 25.6% (n=84) among year 4 (χ^2^_3_= 26.198, *P*<.001).

### Influence of Mass and Social Media on Psychobehavioral Responses

As shown in Table 2, multivariable regression analysis of factors influencing a higher score of mass media exposure showed significant associations with all the HBM constructs. Higher perception of severity (OR 1.33; 95% CI 1.10-1.60), self-efficacy (OR 2.03; 95% CI 1.64-2.52), and perceived control or intention to carry out prevention measures (OR 1.29; 95% CI 1.07-1.56) were significantly associated with a higher mass media exposure score. Lower emotional consequences (OR 1.51; 95% CI 1.25-1.83) and barriers to carry out preventive measures (OR 1.50; 95% CI 1.26-1.84) were also significantly associated with a higher mass media exposure score.

Multivariable regression analysis of factors influencing a higher score of social media exposure showed significant associations with 3 of the HBM constructs. Higher perception of severity (OR 1.41; 95% CI 1.17-1.69), self-efficacy (OR 2.01; 95% CI 1.67-2.58), and perceived control or intention to carry out prevention measures (OR 1.27; 95% CI 1.05-1.53) were significantly associated with a higher social media exposure score. Likewise, lower emotional consequences (OR 1.50; 95% CI 1.24-1.67) and barriers to carry out preventive measures (OR 1.39; 95% CI 1.15-1.67) were also significantly associated with a higher social media exposure score.

A lower anxiety score was significantly associated with higher mass and social media exposure in the univariable analyses; however, the associations were not significant in the multivariable analyses.

**Table 2:** Factors associated with mass media and social media exposure (N=2086).

Variable			Univariate analysis (mass media exposure score 20-24 vs 0-19^a^)		Multivariable logistic regression (mass media exposure score 20-24 vs 0-19^a^)	Univariate analysis (social media exposure score 23-30 vs 0-22^b^)		Multivariable logistic regression (social media exposure score 23-30 vs 0-22^b^)
			High score (20-24) (n=1113)	*P* value	OR^c^ (95%CI)	High score (23-30) (n=1096)	*P* value	OR (95%CI)
**Demographic characteristics**								
	**Age group (years)**							
		18-20	615 (51.4)		—^d^	625 (52.2)		—
		21-22	397 (55.6)	.10	—	377 (52.8)	.92	—
		23-29	101 (57.7)		—	94 (53.7)		—
	**Birthplace**							
		Urban	402 (56.1)	.08	—	395 (55.1)	.09	—
		Rural	711 (51.9)		—	701 (51.2)		—
	**Annual family income (CNY)**							
		<50,000	503 (51.4)		—	497 (50.8)		Ref^e^
		50,000-120,000	421 (54.3)	.19	—	404 (52.1)	.048	1.01 (0.83-1.24)
		>120,000	189 (56.8)		—	195 (58.6)		1.28 (0.98-1.66)
	**Year**							
		1	338 (51.1)		Ref	338 (51.1)		—
		2	234 (47.8)		0.94 (0.73-1.20)	239 (48.8)	.07	—
		3	345 (56.9)	.001	1.30 (1.03-1.64)^f^	333 (55.0)		—
		4 and postgraduate	196 (59.8)		1.31 (0.99-1.74)	186 (56.7)		—
**Health Belief Model**								
	**Perceived susceptibility**							
		Certainly no/probably no/probably yes	1056 (52.8)		Ref	1033 (51.6)		Ref
		Certainly yes	57 (67.1)	.01	1.1 (0.75-1.96)	63 (74.1)	<.001	1.75 (1.05-2.93)
	**Perceived severity**							
		Not at all/slightly serious/serious	524 (47.6)	*<*.001	Ref	509 (46.2)	<.001	Ref
		Very serious	589 (59.8)		1.33 (1.10-1.60)^g^	587 (59.6)		1.41 (1.17-1.69)^h^
	**Perceived self-efficacy**							
		Certainly no/probably no/probably yes	205 (35.3)	*<*.001	Ref	199 (34.3)	*<*.001	Ref
		Certainly yes	908 (60.3)		2.03 (1.64-2.52)^h^	897 (59.6)		2.01 (1.67-2.58)^h^
	**Perceived control or intention to carry out preventive measures**							
		Certainly no/probably no/probably yes	293 (49.9)	.01	Ref	386 (49.0)	.01	Ref
		Certainly yes	720 (55.5)		1.29 (1.07-1.56)^g^	710 (54.7)		1.27 (1.05-1.53)^f^
**Psychological and behavioral response**								
	**Emotional consequences**							
		Scores 5-9	625 (62.3)	*<*.001	1.51 (1.25-1.83)^h^	607 (60.5)	*<*.001	1.50 (1.24-1.67)^h^
		Scores 10-20	488 (45.1)		Ref	489 (45.2)		Ref
	**Barriers to carry out preventive measure**							
		Scores 8-15	618 (62.7)	*<*.001	1.5 (1.26-1.84)^h^	608 (61.7)	*<*.001	1.39 (1.15-1.67)^g^
		Scores 16-32	495 (45.0)		Ref	488 (44.4)		Ref
**Anxiety level**								
	**State-Trait Anxiety Inventory**							
		Score 20-43	731 (56.6)	<.001	1.16 (0.96-1.40)	713 (55.2)	.002	1.11 (0.90-1.34)
		Score 44-80	382 (48.1)		Ref	383 (48.2)		Ref

^a^Hosmer–Lemeshow test, chi-square: 4.749, *P*=.78; Nagelkerke R^2^=0.116.

^b^Hosmer–Lemeshow test, chi-square: 16.804, *P*=.03; Nagelkerke R^2^=0.112.

^c^OR: odds ratio.

^d^Not applicable.

^e^Ref: reference.

^f^*P<*.05.

^g^*P<*.01.

^h^*P<*.001.

## Discussion

### Principal Findings

This study assessed both mass and social media exposure related to COVID-19 and investigated the association between media exposure and HBM constructs, psychological and behavioral responses, and anxiety levels. This study targeted university students, as university students are among the biggest users of the internet and social media [20]. Since this study was conducted when the country was experiencing a decline in COVID-19 cases, it has the advantage of identifying detrimental psychobehavioral factors to provide insight for interventions to prevent a resurgence of infections. Of note, during the data collection period, the nationwide lockdown and movement control had started to ease; nevertheless, schools and universities in China had not yet reopened.

The high mean total exposure score implies that university students have high exposure to both mass media and social media during the COVID-19 outbreak. This finding replicates evidence from previous research, indicating high use of online media (particularly social media) by the younger generation and specifically university students [21-24]. In this study, we also found that university students were exposed to equal amounts of COVID-19–related information from both mass and social media. Both mass and social media were equally used as information sources for the prevention of infection, symptoms, risk, and mode of transmission.

Despite China’s downward COVID-19 trend, the study participants demonstrated a high perceived risk of COVID-19 infection. However, a relatively lower perception of the severity of COVID-19 infection was observed. Many participants also reported high self-efficacy in carrying out recommended prevention measures. During the early phase of the outbreak, the country carried out aggressive public health interventions, such as early detection of cases, contact tracing, and population behavioral changes, which have been reported to have contributed enormously to containing the epidemic [25]. The positive psychobehavioral responses found in this study indicate that population behavioral change interventions have brought about positive behavioral as well as attitudinal changes up to the present time, which is reflected in the success in curbing the spread of the virus to the wider community as observed in the continuous slowdown of COVID-19 cases in China.

The study also found an overall low level of emotional consequences among participants during the off-peak period of the COVID-19 outbreak, as shown by the low mean value of the total emotional consequences score. Despite the low level of emotional consequences, it should be noted that continuous mitigation of the emotional well-being of the public during an infectious disease outbreak is important in controlling transmission [26]. During the severe acute respiratory syndrome (SARS) epidemic, fear and stigma may have instigated people to delay seeking care and remain in the community undetected. Also, a noteworthy finding is that the most prominent emotional consequence found was avoidance behavior, as it was reported by nearly half of the study participants. It is important to note that cognitive avoidance contributes to a delay in taking precautions to prevent the spread of COVID*-*19. The implication of this is that prompt action by the public and immediate seeking of medical care upon suspicion of COVID-19 infection are still needed, regardless of the downward trend.

This study’s participants found minimal difficulty in carrying out preventive measures. The most prominent difficulty encountered was avoiding touching one’s eyes, nose, and mouth; nearly half the participants reported having experienced this difficulty. The importance of refraining from touching one’s eyes, nose, and mouth with unwashed hands to prevent the transmission of COVID-19 has been noted previously [27]. Since habitual face-touching behavior has been commonly reported [28], hand hygiene compliance should be encouraged to avoid this route of transmission. Public health interventions to promote and encourage desirable hand-hygiene–compliant behaviors are crucial even though the outbreak is largely under control.

During the early phase of the pandemic, more than half (53.8%) of the general public in China reported the psychological impact associated with COVID-19 as being moderate or severe [8]. In this study, slightly over one-third (38.1%) of university students reported moderate-to-severe anxiety. Although relatively lower anxiety levels were observed after the peak of the outbreak, our results indicate that COVID-19 is still spurring fear in some parts of society. In the case of the Ebola outbreak, anxiety and depression were still prevalent 1 year after the outbreak, especially among those who had been in quarantine and witnessed death associated with the disease [29]. Findings from this study imply that COVID-19–related anxiety among university students warrants special attention. Therefore, it is suggested that continuous assessment and monitoring of COVID-19–associated mental health issues is essential when students resume their studies on campus. Mental health service provision or psychological intervention services to help students who experience loss of family members or friends to COVID-19 should be encouraged in all universities across the country, especially in Wuhan, China's coronavirus epicentre. Furthermore, the study also found that younger university students were more vulnerable to moderate-to-severe anxiety; more attention from university authorities should be allocated to monitor the mental well-being of these students.

The results of the multivariate analyses of this study provide evidence of the important role of both mass and social media in shaping individual health beliefs using the HBM constructs. Substantial mass media exposure was associated with having a higher perception of illness severity and a higher perceived control or intention to carry out prevention measures. Similarly, social media exposure shapes individual health beliefs using the HBM constructs. However, high social media exposure was associated with all the HBM constructs investigated, except for the perception of risk.

Previous reports have noted that emotional consequences such as fear, stigma, and discrimination during the COVID-19 outbreak among people in China were fuelled by misinformation and unfounded rumors [30]. In our study, multivariate analyses revealed that greater mass and social media exposure were also associated with lower emotional consequences, namely, perception of avoidance, embarrassment, fear, and keeping the infection a secret. This perhaps implies that our study participants were exposed to credible and accurate information from both mass and social media, and hence were not negatively impacted. Of note, the Chinese government implemented viable strategies to counter misinformation and fake news during the pandemic such as immediate removal of fake news in the media and strict penalties for offenders.

The behavioral influence of both mass and social media were evident in this study. More mass and social media exposure was also associated with lower barriers to carrying out prevention practices. The findings imply the importance of continuously providing the public with accurate and credible information through mass and social media to enhance emotional well-being and prevention behaviors. It is also vital for media authorities to ensure the credibility of information shared in during an infectious pandemic to elevate negative psychological impact and enhance prevention behaviors. It has been suggested that quick and targeted interventions oriented to delegitimize sources of fake information in the media are important to reduce negative consequences [31]. As such, the findings of this study provide insights into the importance of developing prompt strategies to counter misinformation.

In short, our findings suggest that both mass and social media are useful means of getting health messages across and contribute to improving psychobehavioral responses to COVID-19. Although traditionally the trustworthiness and authenticity of information sourced from social media in relation to mass media has been an issue of concern, this study demonstrated contrary results. Both mass and social media contributed similarly to favorable psychobehavioral responses to COVID-19.

Interestingly, the univariable analyses also observed that both high levels of mass and social media usage were significantly associated with lower anxiety levels. However, the association was not significant in the multivariable analyses. Our finding contradicts recent findings that reported a high prevalence of mental health problems among the public in China that was positively associated with frequent exposure to social media [14]. Of note, our study participants were medical and health sciences students, and this perhaps implies that they were more proficient at identifying and consuming credible information on social media than the general public. In addition, our study has also demonstrated that students with higher exposure to mass and social media tend to have lower negative emotional consequences and fewer barriers to carrying out prevention measures, which might partly contribute to their lower anxiety level. Thus, their increased social media usage does not result in a higher level of mental health problems. This possibly suggests that the proper use of social media for information purposes is beneficial is shaping psychological and behavioral responses during an infectious disease outbreak.

### Limitations

This study has several limitations that should be considered. The first pertains to the use of convenience sampling and its cross-sectional nature. It cannot, therefore, be used to infer causality. Despite of the recruitment of a large and diverse sample, the relatively high proportion of young participants in this study may introduce a bias toward greater social media usage. Second, the responses were based on self-report and may be subject to recall bias, self-reporting bias, and a tendency to report socially desirable responses. A third limitation is that the participants were medical and health sciences students; this warrants careful interpretation owing to their comprehensive knowledge and attitude about COVID-19 as well as their higher affinity for health information. Next, the associations found in this study should be interpreted with caution as the psychobehavioral responses were obtained during the off-peak period of the COVID-19 outbreak. Despite these limitations*,* the study data contribute tremendously to the understanding of the influence of both mass and social media on psychobehavioral responses to the COVID-19 outbreak in China.

### Conclusions

Higher exposure to both mass and social media related to the COVID-19 outbreak increased positive attitudes in all the domains of the HBM. Emotional* *consequences and behavioral prevention barriers also reduced with higher exposure to both mass and social media. In conclusion, based on our results, both mass and social media are useful means of disseminating health-related information to the public and contribute to improvements in psychobehavioral responses to COVID-19. Our findings imply that university students are proficient at identifying and consuming credible information on social media. With much information circulating on the internet, it is challenging for the public to stay informed with reliable, credible, and trustworthy information from the internet. The general public should be informed about proper online health information seeking during disease outbreaks to avoid detrimental psychological and behavioral impacts that may deter outbreak management and control.

## Abbreviations

COVID-19: coronavirus disease
HBM: Health Belief Model
OR: odds ratio
SARS: severe acute respiratory syndrome
STAI: State-Trait Anxiety Inventory
